# Diagnosis of Pancreatic Neoplasms Using a Novel Method of DNA Methylation Analysis of Mucin Expression in Pancreatic Juice

**DOI:** 10.1371/journal.pone.0093760

**Published:** 2014-04-08

**Authors:** Seiya Yokoyama, Sho Kitamoto, Michiyo Higashi, Yuko Goto, Taro Hara, Dai Ikebe, Taketo Yamaguchi, Yoshifumi Arisaka, Toru Niihara, Hiroto Nishimata, Sadao Tanaka, Kyoichi Takaori, Surinder K. Batra, Suguru Yonezawa

**Affiliations:** 1 Department of Human Pathology, Field of Oncology, Kagoshima University Graduate School of Medical and Dental Sciences, Kagoshima, Kagoshima, Japan; 2 Division of Endoscopy, Chiba Cancer Center, Chiba, Chiba, Japan; 3 Division of Surgical Pathology, Chiba Cancer Center, Chiba, Chiba, Japan; 4 Division of Gastroenterology, Chiba Cancer Center, Chiba, Chiba, Japan; 5 Department of Gastroenterology, Kobe University Graduate School of Medicine, Kobe, Hyogo, Japan; 6 Division of Gastroenterology, Nanpuh Hospital, Kagoshima, Kagoshima, Japan; 7 Division of Surgical Pathology, Nanpuh Hospital, Kagoshima, Kagoshima, Japan; 8 Division of Hepato-Biliary-Pancreatic Surgery and Transplantation, Department of Surgery, Kyoto University Graduate School of Medicine, Kyoto, Kyoto, Japan; 9 Department of Biochemistry and Molecular Biology, Eppley Institute for Research in Cancer and Allied Diseases, University of Nebraska Medical Center, Omaha, Nebraska, United States of America; UCSF/VA Medical Center, United States of America

## Abstract

Mucins (MUC) play crucial roles in carcinogenesis and tumor invasion in pancreatic ductal adenocarcinoma (PDAC) and intraductal papillary mucinous neoplasms (IPMNs). Our immunohistochemistry (IHC) studies have shown a consensus position on mucin expression profiles in pancreatic neoplasms as follows: MUC1-positive but MUC2-negative expression in PDACs; MUC1-negative but MUC2-positive expression in intestinal-type IPMNs (dangerous type); MUC1-negative and MUC2-negative expression in gastric-type IPMNs (safe type); High MUC4 expression in PDAC patients with a poor outcome; and MUC4-positive expression in intestinal-type IPMNs. We also showed that three mucin genes (*MUC1*, *MUC2* and *MUC4*) expression in cancer cell line was regulated by DNA methylation. We have developed a novel ‘methylation-specific electrophoresis (MSE)’ method to analyze the DNA methylation status of mucin genes by high sensitivity and resolution. By using the MSE method, we evaluated pancreatic juice samples from 45 patients with various pancreatic lesions. The results were compared with final diagnosis of the pancreatic lesions including IHC of mucin expression in the paired pancreatic tissues. The results indicated that the DNA methylation status of *MUC1*, *MUC2* and *MUC4* in pancreatic juice matched with the mucin expression in tissue. Analyses of the DNA methylation status of *MUC1*, *MUC2* and *MUC4* were useful for differential diagnosis of human pancreatic neoplasms, with specificity and sensitivity of 87% and 80% for PDAC; 100% and 88% for intestinal-type IPMN; and 88% and 77% for gastric-type IPMN, respectively. In conclusion, MSE analysis of human pancreatic juice may provide useful information for selection of treatment for pancreatic neoplasms.

## Introduction

Patients with pancreatic ductal adenocarcinoma (PDAC) have a poor clinical outcome, despite improvements in diagnosis and treatment methods. Resection at an early stage gives a relatively favorable outcome, but PDACs are diagnosed in an advanced stage in most cases [1]. Indolent neoplasms such as intraductal papillary mucinous neoplasms (IPMNs) also occur in the pancreas [2] and sometimes transform into lesions with an invasive character and a poor outcome [3], [4]. An IPMN is a mucin-producing cystic neoplasm that was first recognized by the World Health Organization (WHO) in 1996 and renamed by the WHO as IPMN in 2000 [5]. Currently, IPMNs are the most common cystic neoplasm of the pancreas, and are classified into gastric, intestinal, pancreatobiliary, and oncocytic types [2], [6]. We have shown that the outcome of intestinal-type IPMN is poorer than that of gastric-type IPMN, although the outcomes for both IPMNs are significantly better than that with PDAC [3], [4]. A recent study also showed that the morphological subtype of IPMN is an independent prognostic factor: *i.e.* patients with gastric-type IPMN have a fair prognosis, those with intestinal-type or oncocytic-type IPMN have a relatively less favorable prognosis, and those with pancreatobiliary-type IPMN have the poorest prognosis [6].

Mucins play crucial roles in diagnostic and prognostic prediction and in carcinogenesis and tumor invasion. MUC1 (pan-epithelial membrane mucin), the first cloned mucin, is an important human tumor antigen, second only to WT1 in cancer antigen pilot prioritization using a ranking based on predefined and preweighted criteria [7]. Our series of immunohistochemistry (IHC) studies has shown a consensus position on mucin expression profiles in pancreatic neoplasms as follows [8], [9]: high expression of MUC1 is observed in PDACs and is related to a poor outcome [10]; intestinal-type IPMNs are MUC1-negative but MUC2 (intestinal secretory mucin)-positive, and sometimes show invasive growth with *de novo* MUC1 expression [3], [4], [11]; gastric-type IPMNs that are MUC1-negative and MUC2-negative have a low potential for malignancy [3], [4]; *de novo* high MUC4 (tracheobronchial mambrane mucin) expression is associated with a poor outcome in patients with PDAC [12]; and MUC4 expression is observed mainly in intestinal-type IPMNs [13].

We have also found that the methylation status, mRNA expression, and mucin core protein expression were well correlated with each other for MUC1, MUC2, and MUC4 in cancer cell lines [14], [15], [16], [17]. In addition, we have developed a novel DNA methylation analysis method ‘methylation specific electrophoresis (MSE, international patent open: WO 2011/132798)’. The MSE method greatly decreases the amount of input DNA and has high sensitivity, although conventional analytical methods for DNA methylation require a large amount of DNA and have low sensitivity. The lower detection limit for distinguishing different methylation status is under 0.1% and the detectable minimum amount of DNA is 20 pg, which can be obtained from only a few cells, and has high resolution [18]. Application of this MSE method in analyses of the epigenetic status of *MUC1*, *MUC2* and *MUC4* in pancreatic juice may be useful for early detection of pancreatic lesion, as further investigated in the current study.

## Materials and Methods

### Cell Lines

Human pancreatic carcinoma cell line HPAF II and Human colon adenocarcinoma cell lines Caco2 and LS174T were obtained from the American Type Culture Collection. HPAF II, Caco2 and LS174T cells were cultured in Eagle’s minimum essential medium (Sigma, St. Louis, MO, USA). The media was supplemented with 10% fetal bovine serum (Invitrogen, Minatoku, Tokyo, Japan) and 100 U/mL of penicillin/100 μg/mL of streptomycin (Sigma). Cell lines with high and low methylation of *MUC1* (Caco2 and LS174T), *MUC2* (HPAF II and LS174T) and *MUC4* (Caco2 and LS174T) were used as control standards in the MSE analysis.

### Clinical Samples

#### Pancreatic tissues

As a basic experiment for the analysis of pancreatic juice, we aimed to examine the relationship between the extent of DNA methylation of mucin genes and the expression level of mRNA in paired pancreatic tissues. Tissue blocks (about 2×2×2 mm) were obtained from neoplastic and non-neoplastic areas of surgically resected fresh specimens of 17 PDACs.

#### Pancreatic juice

After completion of endoscopic retrograde pancreatography, pancreatic juice was collected using endoscopic nasopancreatic drainage, pancreatic stenting, a bottle-shaped metal tip endoscopic retrograde cholangiopancreatography catheter (5 Fr; MTW Endoskopie Inc., Wesel, Germany) [19], [20].

#### Ethics statement

The study was conducted in accordance with the guiding principles of the Declaration of Helsinki. Collection of samples was approved by the ethical committees of each hospital (Ethical committees of Kagoshima University Hospital, Chiba Cancer Center Hospital, Osaka Medical College Hospital, Nanpuh Hospital and Kyoto University Hospital), and informed written consent was obtained from each patient. All studies using human materials in this article were approved by the ethical committee of Kagoshima University Hospital (revised 20–82 and revised 22–127).

### Extraction and Quantification of mRNA

Total RNA was extracted from cell lines, human pancreatic tissues and pancreatic juices using a RNeasy Mini kit (QIAGEN, Chuo-ku, Tokyo, Japan). Total RNA (1 μg) was reverse transcribed with a High Capacity RNA-to-cDNA Kit (Applied Biosystems, Foster City, CA, USA). Real-time reverse transcription–PCR was performed on a ABI PRISM 7000 Sequence Detection System using SYBR Green PCR Master Mix (Applied Biosystems). Gene expression was normalized to the β-actin mRNA level in each sample. Primer sets are shown in Table S1.

### Extraction of DNA and Bisulfite Modification

DNA from cell lines, pancreatic tissue, and pancreatic juice was extracted using a DNeasy Tissue System (QIAGEN). Bisulfite modification of the genomic DNA was carried out using an Epitect Bisulfite Kit (QIAGEN). Purification of PCR products was carried out using a Wizard SV Gel and PCR Clean-Up System (Promega KK, Chuo-ku, Tokyo, Japan).

### MSE Analysis

MSE analysis was performed as follows. In the preparation of the samples step, the target DNA fragments were amplified by nested PCR approach using bisulfite treated DNA. The using primer sets were shown in Table S1. In the electrophoresis step, the amplicon was applied to the D-Code system (BioRad Laboratories, Hercules, CA, USA) using polyacrylamide gel with linear denaturant gradient at 60°C, 70 V for 14 h. The detailed informations of MSE method were described in our previous study [18]. The band intensity was measured by Image J software (National Institutes of Health <http://rsb.info.nih.gov/ij/>). The unmethylation index (U-index) was calculated as U-index  =  (highest band intensity/total band intensity)_ sample_/(highest band intensity/total band intensity)_ basal cell line_×100. Thus, the U-index in each sample was normalized using data from a hypomethylated cell line.

### Statistical Analysis

Data were analyzed using the “R” computing environment [21]. The normality of the data distribution was evaluated by Kolmogorov-Smirnov test. Differences between groups were analyzed by Student t-test or Welch t-test. A nonparametric test of group difference was performed by Mann–Whitney U test. Correlations were tested using single regression analysis. Quadratic discrimination analysis and canonical discriminant analysis were performed with the R add-on MASS package [22]. The threshold value and area under the curve (AUC) were calculated by receiver operating characteristics (ROC) curve analysis [23]. A p value<0.05 was considered statistically significant.

### Immunohistochemical Staining

IHC was performed in cut sections of pancreatic tumors using anti-MUC1 monoclonal antibody (MAb) clone 014E (MAb MUC1/014E, generated by one of us, Suguru Yonezawa) [24]; anti-MUC2 MAb clone Ccp58 (MAb MUC2/Ccp58, Novocastra Reagents, Leica Biosystems, Newcastle Upon Tyne, UK) and anti-MUC4 MAb clone 8G7 (MAb MUC4/8G7, generated by one of us, Surinder K. Batra) [25], using the immunoperoxidase method. Antigen retrieval was performed using CC1 antigen retrieval buffer (pH 8.5, EDTA, 37°C, 30 min; Ventana Medical Systems, Tucson, AZ, USA) for all sections. Following incubation with the primary antibodies (MAb MUC1/014E diluted 1∶5, 37°C, 32 min; MAb MUC2/Ccp58 diluted 1∶200, 37°C, 32 min; MAb MUC4/8G7 diluted 1∶3000, 37°C, 32 min) in phosphate buffered saline pH 7.4 (PBS) with 1% bovine serum albumin (BSA), sections were stained on a Benchmark XT automated slide stainer using a diaminobenzidine detection kit (UltraView DAB, Ventana Medical Systems). The control staining using normal mouse serum or PBS-BSA instead of the primary antibodies always showed no reaction.

## Results

### DNA Methylation Status and Expression Level of mRNA in PDAC Tissues

To examine the relationship between the extent of DNA methylation of mucin genes and the expression level of mRNA in paired pancreatic tissues, we evaluated 34 tissue samples (17 paired, neoplastic and non-neoplastic areas of PDAC specimens).

**Figure 1:**
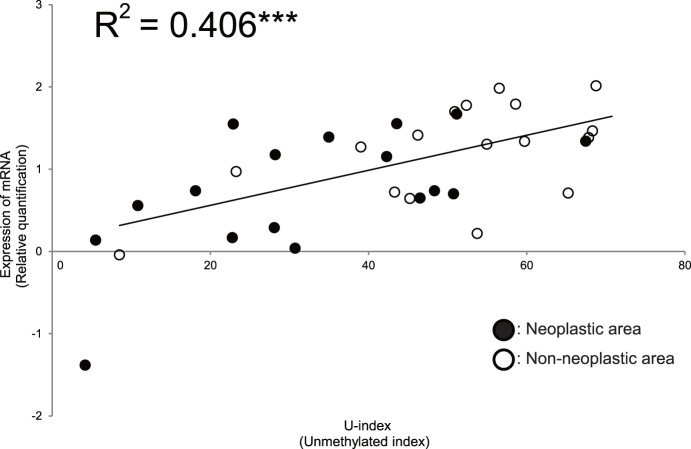
Expression level of MUC1 mRNA and methylation status in pancreatic tissue of PDAC specimens. Correlation analysis of mRNA levels and extent of DNA methylation. The *MUC1* U-index showed a strong correlation with the MUC1 mRNA level (R^2^ = 0.406, P<0.001). Relative mRNA expression was calculated based on the expression level of MUC1 in a human pancreatic cell line (Panc1). The U methylation index (U-index) was normalized using a cell line (LS-174T) with low methylation.

A plot of the U-index for *MUC1* against the mRNA level for MUC1 showed a significant correlation (R^2^ = 0.406, P<0.001) (Figure 1). This result indicates that the extent of DNA methylation status of *MUC1* is a trigger for regulation of expression of MUC1 mRNA in pancreatic tissue.

A plot of the U-index for *MUC2* against the mRNA level for MUC2 showed no significant correlation (data not shown). A plot of the U-index for *MUC4* against the mRNA level for MUC4 showed no significant correlation, either (data not shown). However, as shown in the following paragraph, the DNA methylation status of *MUC2* and *MUC4* could be applied in the analysis of pancreatic juice.

### Correlation between DNA Methylation Status in Pancreatic Juice and Mucin Expression

**Figure 2:**
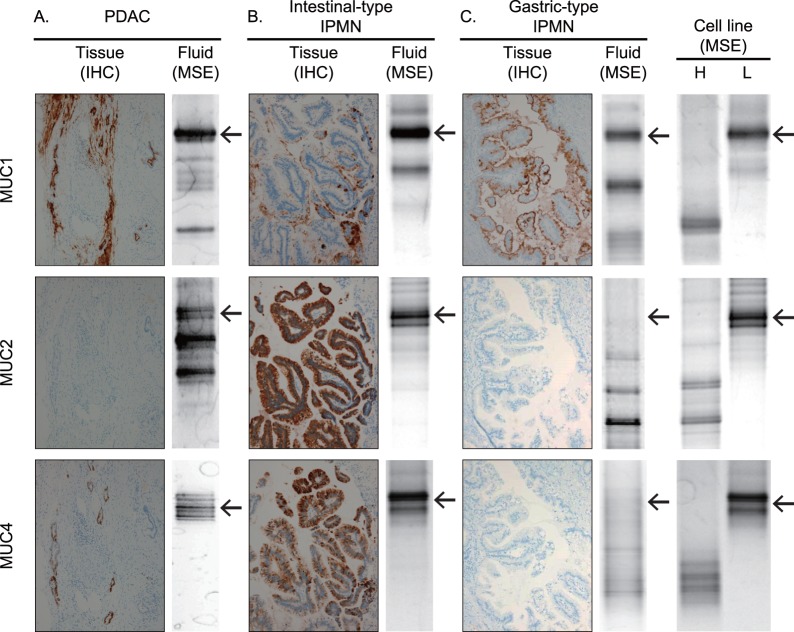
Correlations between results of pancreatic tissue analysis and fluid analysis. A: Pancreatic juice from patients with PDAC showed a unmethylated *MUC1* (U-index: 65.1 point), methylated *MUC2* (U-index: 42.7 point), and unmethylated *MUC4* (U-index: 69.4 point) in MSE analysis, and paired pancreatic tissues were MUC1-positive, MUC2-negative and MUC4-positive in immunohistochemistry (IHC). B: Pancreatic juice from patients with intestinal-type IPMN showed unmethylated *MUC1* (U-index: 76.7 point), unmethylated *MUC2* (U-index: 96.0 point) and unmethylated *MUC4* (U-index: 92.2 point)in MSE analysis, and paired pancreatic tissues were MUC1- positive, MUC2- positive and MUC4-positive in IHC. C: Pancreatic juice from patients with gastric-type IPMN showed unmethylated *MUC1*(U-index: 22.8 point) and methylated *MUC2* (U-index: 4.5 point) and methylated *MUC4* (U-index: 46.4 point) in MSE analysis, and paired pancreatic tissues were MUC1-positive and MUC2- negative and MUC4-negative in IHC. Cell line results of MSE analysis for MUC1, H: highly methylated (Caco2), L: low methylation (LS-174T); for MUC2, H: highly methylated (HPAF II), L: low methylation (LS-174T); for MUC4, H: highly methylated (Caco2), L: low methylation (LS-174T). Arrows indicate the highest band using for calculation of U-index.

Representative cases of comparison of the DNA methylation status using MSE of pancreatic juice and expression of mucins examined by IHC in paired pancreatic tissues from PDAC, intestinal-type IPMN and gastric-type IPMN are shown in Figure 2. Pancreatic juice from patients with PDAC showed unmethylated *MUC1*, methylated *MUC2*, and unmethylated *MUC4* in MSE analysis, and paired pancreatic tissues were MUC1-positive, MUC2-negative and MUC4-positive (Figure 2A). Pancreatic juice from patients with intestinal-type IPMN showed unmethylated *MUC1*, unmethylated *MUC2* and unmethylated *MUC4*, and paired pancreatic tissues were MUC1-positive, MUC2-positive and MUC4-positive (Figure 2B). Pancreatic juice from patients with gastric-type IPMN showed unmethylated *MUC1*, methylated *MUC2* and methylated *MUC4*, and paired pancreatic tissues were MUC1-positive and MUC2-negative and MUC4-negative (Figure 2C). These results indicate that the DNA methylation status of the three mucin genes (*MUC1*, *MUC2* and *MUC4*) in pancreatic juice matches with the expression level of the three mucins (MUC1, MUC2 and MUC4) in tissue. Thus, MSE analysis of pancreatic juice may be useful for assessment of mucin expression levels.

### Differences in DNA Methylation among Neoplastic Lesions in Pancreatic Juice Analysis

**Figure 3:**
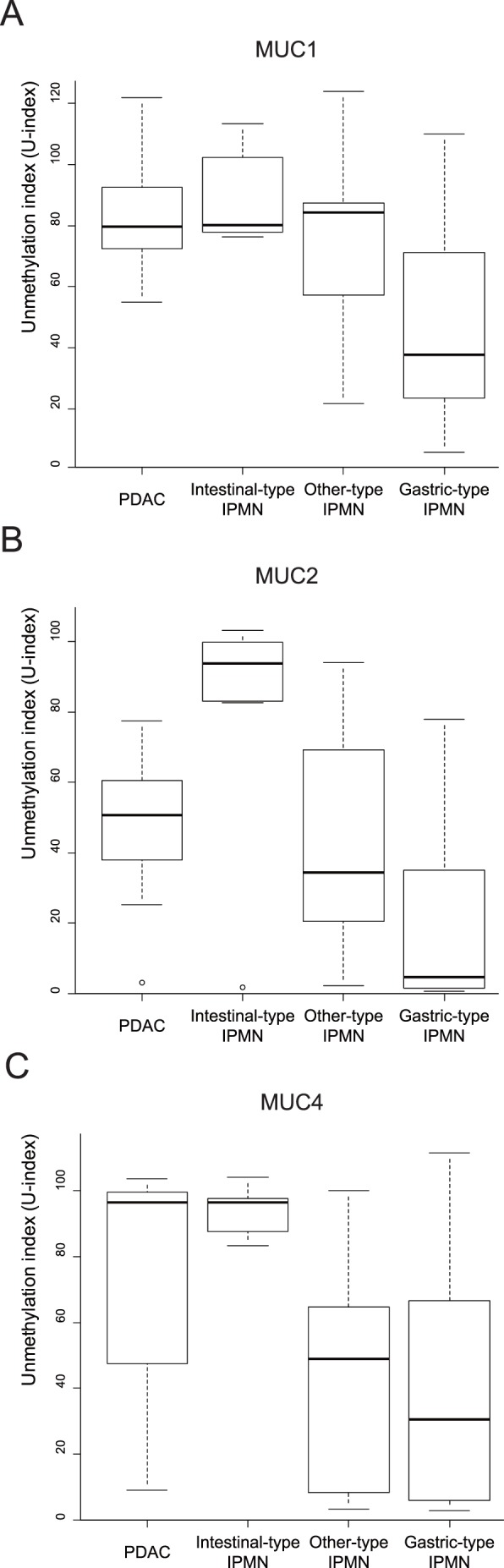
Methylation status of mucin genes obtained from pancreatic juice in each disease type. For all three mucins, the U-index was normalized to a cell line with low methylation (LS-174T). A: For MUC1, gastric-type IPMN had a significantly lower U-index than PDAC and other IPMN types; that is, the MUC1 promoter was most methylated (hypermethylated) in gastric-type IPMN. B: For MUC2, intestinal-type IPMN had a significantly higher U-index than PDAC and other IPMN types; that is, the MUC2 promoter was most unmethylated (hypomethylated) in intestinal-type IPMN. C: For MUC4, PDAC and intestinal-type IPMN had a significantly higher U-index than other-type IPMN and gastric-type IPMN.

To examine differences in DNA methylation of *MUC1*, *MUC2* and *MUC4* among pancreatic neoplastic lesions, we evaluated pancreatic juice samples from 15 patients with PDAC, 11 patients with gastric-type IPMN, 8 with intestinal-type IPMN, 9 with other IPMN types, and 2 non-neoplastic pancreases (Table 1). The promoter methylation status of the three mucins was detected by MSE and the U-index was calculated using the band intensity. Interestingly, pancreatic juices obtained from the 2 non-neoplastic pancreases were similar to those for gastric-type IPMN (data not shown). The median U-index, 95% confidence interval and p values in a Kolmogorov-Smirnov test are shown in Table S2. For MUC1, gastric-type IPMN had a significantly lower U index (P>0.001) compared to other diseases, including intestinal-type IPMN and PDAC (Figure 3A). The area under the curve (AUC) for distinguishing gastric-type IPMN from other neoplasms was 0.803 (ROC curve shown in Figure S1A; U-index threshold of 44.65 points). For MUC2, the intestinal-type IPMN had a significantly higher U-index (P>0.001) compared to the other neoplasms, including gastric-type IPMN and PDAC, and the U-index for gastric-type IPMN was significantly lower (P = 0.002) than that for PDAC (Figure 3B). The AUC for distinguishing intestinal-type IPMN from other neoplasms was 0.882 (ROC curve shown in Figure S1B; U-index threshold of 82.71 points). For MUC4, gastric-type IPMN had a significantly lower U-index (P = 0.018) than intestinal-type IPMN (Figure 3C). The AUC for distinguishing gastric-type IPMN from intestinal-type IPMN was 0.740 (ROC curve shown in Figure S1C; U-index threshold of 83.22 points). The threshold values, AUCs, and P values are summarized in Table 2.

**Table 1:** Origin of pancreatic juice samples.

Disease state	Number
PDAC	15
Intestinal-type IPMN	8
Other-type IPMN	
Pancreatobiliary-type IPMN	4
Intestinal-type IPMN with Colloid Carcinoma	3
Oncocytic-type IPMN	1
Intraductal tubulopapillary neoplasm	1
Gastric-type IPMN	11
Non-neoplastic pancreas	2
Total	45

PDAC: pancreatic ductal adenocarcinoma, IPMN: intraductal papillary mucinous neoplasm.

**Table 2:** Summary of P values in T tests, AUCs and threshold values.

1. MUC1													
PDAC vs IPMN-IN			PDAC vs IPMN-Oh				PDAC vs IPMN-GA				PDAC vs Other		
P	AUC	Th.	P	AUC	Th.		P	AUC	Th.		P^a^	AUC	Th.
0.355	0.617	76.25	0.514	0.526	55.03		0.001	0.815	55.03		0.057	0.613	55.03
			IPMN-IN vs IPMN-Oh				IPMN-IN vs IPMN-GA				IPMN-IN vs Other		
			P	AUC	Th.		P	AUC	Th.		P	AUC	Th.
			0.256	0.611	76.25		0.002	0.837	76.25		0.064	0.693	76.25
							IPMN-Oh vs IPMN-GA				IPMN-Oh vs Other		
							P	AUC	Th.		P	AUC	Th.
							0.050	0.752	44.65		0.701	0.556	84.27
											IPMN-GA vs Other		
											P	AUC	Th.
											<0.001	0.803	44.65
**2. MUC2**													
PDAC vs IPMN-IN			PDAC vs IPMN-Oh			PDAC vs IPMN-GA				PDAC vs Other			
P	AUC	Th.	P	AUC	Th.	P		AUC	Th.	P^a^		AUC	Th.
0.005	0.875	82.71	0.457	0.622	37.41	0.002		0.821	35.99	0.522		0.576	35.99
			IPMN-IN vs IPMN-Oh			IPMN-IN vs IPMN-GA				IPMN-IN vs Other			
			P	AUC	Th.	P		AUC	Th.	P		AUC	Th.
			0.018	0.833	82.71	<0.001		0.923	82.71	<0.001		0.882	82.71
						IPMN-Oh vs IPMN-GA				IPMN-Oh vs Other			
						P		AUC	Th.	P		AUC	Th.
						0.106		0.735	8.46	0.687		0.540	37.41
										IPMN-GA vs Other			
										P		AUC	Th.
										0.001		0.822	8.46
**3. MUC4**													
PDAC vs IPMN-IN			PDAC vs IPMN-Oh			PDAC vs IPMN-GA				PDAC vs Other			
P^b^	AUC	Th.	P^b^	AUC	Th.	P^b^		AUC	Th.	P^b^		AUC	Th.
0.825	0.533	98.20	0.030	0.770	68.98	0.065		0.708	92.54	0.052		0.680	92.54
			IPMN-IN vs IPMN-Oh			IPMN-IN vs IPMN-GA				IPMN-IN vs Other			
			P	AUC	Th.	P		AUC	Th.	P		AUC	Th.
			0.019	0.792	83.22	0.019		0.740	83.22	0.061		0.642	83.22
						IPMN-Oh vs IPMN-GA				IPMN-Oh vs Other			
						P		AUC	Th.	P^b^		AUC	Th.
						0.980		0.504	48.98	0.111		0.676	68.98
										IPMN-GA vs Other			
										P^b^		AUC	Th.
										0.101		0.659	48.98

a Welch T test,

b Mann-Whitney U test, PDAC: pancreatic ductal adenocarcinoma, IPMN: intraductal papillary mucinous neoplasm, IPMN-IN: intestinal-type IPMN, IPMN, IPMN-Oh: other-type IPMN, IPMN-GA: gastric-type IPMN, Th.: Threshold value.

### Distinction of Pancreatic Neoplastic Lesions Based on Aberrant Methylation of Three Mucins

A predictive model for identification of pancreatic disease was constructed using the U-indexes of MUC1, MUC2 and MUC4 based on quadratic (Table 3) or canonical (Table 4) discriminant analysis. The model based on quadratic discrimination analysis had specificity and sensitivity of 87% and 80% for PDAC; 100% and 88% for intestinal-type IPMN (dangerous type); and 88% and 77% for gastric-type IPMN (safe type) and non-neoplastic case. With canonical discriminant analysis, the model had a specificity and sensitivity of 77% and 73% for PDAC; 95% and 88% for intestinal-type IPMN; and 91% and 69% for gastric-type IPMN. These data are summarized in Table S3. The accuracies of the quadratic and canonical discriminant analyses were 76% and 64%, respectively, in examination of 45 pancreatic juice samples. Thus, quadratic discrimination analysis was more suitable for construction of the predictive model for pancreatic disease type using analysis of pancreatic juice.

**Table 3:** Summary of predictive model findings for disease type in Quadratic discrimination analysis.

		Probability of prediction					
Final diagnosis	Setting of disease type	PDAC	IPMN-IN	Other	IPMN-GA	Predicted disease type	Accuracy
PDAC	PDAC	50.0%	35.6%	8.1%	6.3%	PDAC	yes
PDAC	PDAC	71.9%	0.0%	2.7%	25.4%	PDAC	yes
PDAC	PDAC	70.2%	0.0%	5.4%	24.5%	PDAC	yes
PDAC	PDAC	48.3%	0.0%	7.7%	44.0%	PDAC	yes
PDAC	PDAC	75.4%	0.0%	2.8%	21.8%	PDAC	yes
PDAC	PDAC	77.2%	0.4%	5.0%	17.4%	PDAC	yes
PDAC	PDAC	77.2%	0.0%	2.6%	20.2%	PDAC	yes
PDAC	PDAC	77.2%	1.7%	5.8%	15.3%	PDAC	yes
PDAC	PDAC	46.8%	9.0%	21.8%	22.3%	PDAC	yes
PDAC	PDAC	71.0%	0.0%	2.8%	26.2%	PDAC	yes
PDAC	PDAC	73.7%	1.5%	7.2%	17.7%	PDAC	yes
PDAC	PDAC	57.2%	3.1%	35.3%	4.4%	PDAC	yes
PDAC	PDAC	32.7%	7.2%	56.7%	3.4%	Other	-
PDAC	PDAC	45.2%	0.6%	53.9%	0.3%	Other	-
PDAC	PDAC	14.3%	0.3%	28.7%	56.7%	IPMN-GA	-
IPMN-IN	IPMN-IN	24.1%	65.6%	7.0%	3.3%	IPMN-IN	yes
IPMN-IN	IPMN-IN	34.5%	52.5%	9.0%	4.0%	IPMN-IN	yes
IPMN-IN	IPMN-IN	2.1%	91.1%	6.0%	0.8%	IPMN-IN	yes
IPMN-IN	IPMN-IN	6.5%	75.4%	17.8%	0.3%	IPMN-IN	yes
IPMN-IN	IPMN-IN	0.5%	95.0%	4.3%	0.2%	IPMN-IN	yes
IPMN-IN	IPMN-IN	0.1%	89.2%	10.6%	0.0%	IPMN-IN	yes
IPMN-IN	IPMN-IN	0.4%	94.3%	5.0%	0.2%	IPMN-IN	yes
IPMN-IN	IPMN-IN	1.6%	37.5%	58.6%	2.4%	Other	-
IPMN-PB	Other	30.3%	0.9%	68.5%	0.3%	Other	yes
IPMN-PB	Other	0.4%	6.7%	86.2%	6.7%	Other	yes
IPMN-PB	Other	11.9%	0.1%	87.9%	0.1%	Other	yes
IPMN-PB	Other	38.8%	0.3%	18.2%	42.7%	IPMN-GA	-
IPMN-IN with CC	Other	5.9%	41.1%	50.6%	2.3%	Other	yes
IPMN-IN with CC	Other	72.5%	1.2%	7.5%	18.8%	PDAC	-
IPMN-IN with CC	Other	12.9%	0.0%	15.5%	71.6%	IPMN-GA	-
IPMN-On	Other	1.2%	0.0%	7.7%	91.1%	IPMN-GA	-
ITPN	Other	10.8%	16.3%	72.8%	0.2%	Other	yes
IPMN-GA	IPMN-GA	1.2%	0.0%	8.0%	90.8%	IPMN-GA	yes
IPMN-GA	IPMN-GA	2.2%	0.0%	7.5%	90.3%	IPMN-GA	yes
IPMN-GA	IPMN-GA	0.1%	0.0%	6.1%	93.8%	IPMN-GA	yes
IPMN-GA	IPMN-GA	7.2%	0.0%	16.4%	76.4%	IPMN-GA	yes
IPMN-GA	IPMN-GA	5.7%	0.0%	11.7%	82.7%	IPMN-GA	yes
IPMN-GA	IPMN-GA	0.4%	0.0%	21.7%	77.9%	IPMN-GA	yes
IPMN-GA	IPMN-GA	7.5%	0.0%	15.8%	76.7%	IPMN-GA	yes
IPMN-GA	IPMN-GA	44.1%	0.0%	11.0%	45.0%	IPMN-GA	yes
IPMN-GA	IPMN-GA	61.3%	0.4%	8.8%	29.5%	PDAC	-
IPMN-GA	IPMN-GA	66.7%	11.7%	9.3%	12.3%	PDAC	-
IPMN-GA	IPMN-GA	69.4%	2.3%	9.5%	18.8%	PDAC	-
Pancreatitis	IPMN-GA	12.8%	0.3%	26.1%	60.8%	IPMN-GA	yes
NL	IPMN-GA	1.4%	0.0%	8.0%	90.6%	IPMN-GA	yes

PDAC: pancreatic ductal adenocarcinoma, IPMN: intraductal papillary mucinous neoplasm, IPMN-IN: intestinal-type IPMN, IPMN-PB: pancreatobiliary-type IPMN, IPMN-IN with CC: intestinal-type IPMN with colloid carcinoma, IPMN-On: oncocytic-type IPMN, ITPN: intraductal tubulopapillary neoplasm, IPMN-GA: gastric-type IPMN, NL: no lesion, Other: Other-type IPMN.

**Table 4:** Summary of predictive model findings for disease type in Canonical discriminant analysis.

		Probability of prediction					
Final diagnosis	Setting of disease type	PDAC	IPMN-IN	Other	IPMN-GA	Predicted disease type	Accuracy
PDAC	PDAC	48.6%	33.8%	15.3%	2.4%	PDAC	yes
PDAC	PDAC	54.2%	1.7%	4.2%	39.9%	PDAC	yes
PDAC	PDAC	61.0%	11.1%	8.3%	19.6%	PDAC	yes
PDAC	PDAC	48.9%	1.4%	10.2%	39.5%	PDAC	yes
PDAC	PDAC	63.9%	5.0%	6.7%	24.4%	PDAC	yes
PDAC	PDAC	63.8%	11.4%	13.6%	11.2%	PDAC	yes
PDAC	PDAC	73.0%	4.1%	8.1%	14.7%	PDAC	yes
PDAC	PDAC	63.4%	13.8%	15.1%	7.7%	PDAC	yes
PDAC	PDAC	64.5%	13.8%	19.5%	2.2%	PDAC	yes
PDAC	PDAC	53.5%	1.5%	4.3%	40.8%	PDAC	yes
PDAC	PDAC	58.2%	20.9%	10.9%	9.9%	PDAC	yes
PDAC	PDAC	18.6%	11.0%	56.0%	14.5%	Other	-
PDAC	PDAC	18.4%	30.1%	41.2%	10.3%	Other	-
PDAC	PDAC	14.4%	11.6%	71.4%	2.6%	Other	-
PDAC	PDAC	25.7%	0.7%	22.4%	51.2%	IPMN-GA	-
IPMN-IN	IPMN-IN	38.9%	45.3%	12.8%	2.9%	IPMN-IN	yes
IPMN-IN	IPMN-IN	41.4%	42.8%	14.5%	1.3%	IPMN-IN	yes
IPMN-IN	IPMN-IN	25.3%	63.0%	8.7%	2.9%	IPMN-IN	yes
IPMN-IN	IPMN-IN	13.0%	77.8%	9.0%	0.3%	IPMN-IN	yes
IPMN-IN	IPMN-IN	18.8%	72.1%	7.0%	2.1%	IPMN-IN	yes
IPMN-IN	IPMN-IN	8.4%	84.5%	6.3%	0.8%	IPMN-IN	yes
IPMN-IN	IPMN-IN	17.8%	72.5%	7.6%	2.2%	IPMN-IN	yes
IPMN-IN	IPMN-IN	23.2%	1.0%	61.9%	13.8%	Other	-
IPMN-PB	Other	14.5%	8.2%	74.8%	2.5%	Other	yes
IPMN-PB	Other	60.4%	2.2%	33.8%	3.6%	PDAC	-
IPMN-PB	Other	11.6%	6.7%	80.2%	1.4%	Other	yes
IPMN-PB	Other	45.1%	1.8%	21.1%	32.0%	PDAC	-
IPMN-IN with CC	Other	18.9%	51.5%	22.1%	7.6%	IPMN-IN	-
IPMN-IN with CC	Other	58.0%	20.7%	10.6%	10.7%	PDAC	-
IPMN-IN with CC	Other	19.9%	0.8%	15.9%	63.4%	IPMN-GA	-
IPMN-On	Other	8.2%	0.3%	7.2%	84.3%	IPMN-GA	-
ITPN	Other	8.5%	76.9%	14.1%	0.5%	IPMN-IN	-
IPMN-GA	IPMN-GA	6.9%	0.1%	1.0%	92.0%	IPMN-GA	yes
IPMN-GA	IPMN-GA	10.2%	0.2%	4.5%	85.0%	IPMN-GA	yes
IPMN-GA	IPMN-GA	3.2%	0.0%	0.7%	96.1%	IPMN-GA	yes
IPMN-GA	IPMN-GA	14.5%	0.5%	16.2%	68.8%	IPMN-GA	yes
IPMN-GA	IPMN-GA	15.2%	0.3%	9.2%	75.4%	IPMN-GA	yes
IPMN-GA	IPMN-GA	12.3%	2.4%	5.9%	79.5%	IPMN-GA	yes
IPMN-GA	IPMN-GA	13.7%	0.9%	17.3%	68.1%	IPMN-GA	yes
IPMN-GA	IPMN-GA	80.6%	0.7%	5.2%	13.5%	PDAC	-
IPMN-GA	IPMN-GA	49.9%	6.4%	17.1%	26.7%	PDAC	-
IPMN-GA	IPMN-GA	52.4%	14.6%	22.6%	10.4%	PDAC	-
IPMN-GA	IPMN-GA	55.3%	28.8%	8.3%	7.7%	PDAC	-
Pancreatitis	IPMN-GA	17.4%	0.7%	24.9%	57.0%	IPMN-GA	yes
NL	IPMN-GA	8.6%	0.3%	7.3%	83.7%	IPMN-GA	yes

PDAC: pancreatic ductal adenocarcinoma, IPMN: intraductal papillary mucinous neoplasm, IPMN-IN: intestinal-type IPMN, IPMN-PB: pancreatobiliary-type IPMN, IPMN-IN with CC: intestinal-type IPMN with colloid carcinoma, IPMN-On: oncocytic-type IPMN, ITPN: intraductal tubulopapillary neoplasm, IPMN-GA: gastric-type IPMN, NL: no lesion, Other: Other-type IPMN.

## Discussion

The accumulating evidences suggested that the DNA methylation in body fluids (e.g., blood, saliva) can be promising biomarkers for various types of cancer [26], [27], [28]. Previous studies also showed that the importance of DNA methylation (such as cyclin D2, ppENK, NPTX2) in pancreatic juice for the diagnosis of pancreatic neoplasms [29], [30]. In our present study, analyses of the DNA methylation status of *MUC1*, *MUC2* and *MUC4* in pancreatic juices were useful for differential diagnosis of human pancreatic neoplasms *i.e.* PDAC, intestinal-type IPMN and gastric-type IPMN, with high specificity and sensitivity.

In analyses of pancreatic neoplastic and non-neoplastic tissues of PDAC samples in this study, we found a strong relationship between the mRNA expression level and DNA methylation status for *MUC1*. This is similar to the results in pancreatic cancer cell lines in our previous study [14] and suggests that DNA methylation has a key role in *MUC1* regulation in human pancreatic tissue. Thus, evaluation of the DNA methylation status of *MUC1* can provide important information for diagnosis of human pancreatic neoplasms. We have reported that MUC2 was not expressed in PDAC and/or non-neoplastic pancreas [8], [9], [10], [31]. Similarly, PDAC and non-neoplastic pancreas showed low expression level of MUC2 mRNA (data not shown). Thus, we could not examine the correlation between DNA methylation status (U index) and expression level of mRNA. In MUC4, PDAC showed higher U index (hypomethylation status of DNA) than paired non-neoplastic area (data not shown). However, significant correlation was not found between DNA methylation status (U index) and expression level of mRNA. This result suggests that other factors affect MUC4 expression. Although there was no relationship between the mRNA expression level and DNA methylation status for *MUC2* and *MUC4* in the tissue samples, the DNA methylation status of *MUC2* and *MUC4* could be applied in the analysis of pancreatic juice as follows.

Since MUC1, MUC2 and MUC4 are key mucins in pathological diagnosis of pancreatic neoplasms [8], [9], [13], [31], our goal is to apply DNA methylation analysis of the three mucin genes using pancreatic juice for early diagnosis of these neoplasms. For this reason, we investigated the DNA methylation status of *MUC1*, *MUC2* and *MUC4* in 45 samples of pancreatic juice collected from patients with PDAC, intestinal-type IPMN, gastric-type IPMN, other-type IPMN and non-neoplastic pancreas.

MSE showed that gastric-type IPMNs have a significantly lower U-index for *MUC1* than other pancreatic neoplasms, indicating that MSE of *MUC1* is useful to identify gastric-type IPMNs. MSE also showed that intestinal type-IPMNs have a significantly higher U-index for *MUC2* compared to other pancreatic neoplasms and that this can be used to identify intestinal-type IPMNs. Interestingly, the *MUC2* analysis also showed a significant difference in methylation status between PDAC and intestinal-type IPMN, and between PDAC and gastric-type IPMN. Such results may provide a diagnostic clue for PDAC. In addition, analysis of *MUC4* using MSE may allow intestinal-type IPMN to be distinguished from gastric-type IPMN. The DNA methylation status of *MUC1*, *MUC2* and *MUC4* in the MSE analysis also matched the expression profiles of the mucin proteins established in our previous studies [3], [4], [8], [9], [10], [12], [13], [31], [32], [33], [34].

Differentiation of gastric-type IPMN (usually a safe type with a favorable outcome) from intestinal-type IPMN (a dangerous type with progression to colloidal carcinoma) by MSE clearly has a major clinical benefit. MSE also allows classification of other pancreatic lesions, including pancreatobiliary-type IPMN and oncocytic-type IPMN, which sometimes overlap with gastric-type IPMN or intestinal-type IPMN [35]. Most gastric-type IPMNs do not require surgery, whereas the other IPMNs usually do need surgical removal [36]. Thus, there may be a significant clinical benefit of MSE analysis of mucin genes using pancreatic juice because this analysis can differentiate IPMNs requiring surgical removal from those that can be treated conservatively with follow-up. Recently, development of PDAC derived from gastric-type IPMN was reported [37]. In the cases of the present study, there is one case of advanced PDAC derived from gastric-type IPMN. The result of MSE analysis of that case showed a pattern of PDAC. Thus, we could differentiate gastric-type IPMN with progression to PDAC, which needs surgical removal, from pure gastric-type IPMN, which does not need surgical removal, by MSE analysis of pancreatic juice.

Pancreatic juice cytology with MUC staining is highly reliable for identifying the preoperative histological subtype of IPMN [20], but cannot be applied to pancreatic juice containing no cells. In contrast, MSE can be used with pancreatic juice containing only DNA fragments. Cells and proteins are easily degraded in pancreatic juice due to the presence of strong digestive enzymes, but DNA fragments may still be present. However, there are many variables between the DNA methylation status as the starting point of mucin synthesis and the final protein product detected by mucin IHC, including the effects of transcription factors, splicing variants, post-transcriptional regulation including microRNAs, and glycosylation. Despite these variables, our MSE analyses of *MUC1*, *MUC2* and *MUC4* in human pancreatic juice showed high sensitivity and specificity for differentiation among PDAC, gastric-type IPMN, intestinal-type IPMN and other-type IPMN.

These findings suggest that MSE analysis of human pancreatic juice can provide useful information for selection of treatment methods for pancreatic neoplasms. Diagnosis can be made using this approach alone, but a combination of MSE analysis with imaging such as ultrasound, computed tomography and magnetic resonance imaging and also with pancreatic juice cytology with MUC staining may permit early differential diagnosis and treatment of pancreatic neoplasms.

## Supporting Information

Figure S1 A: ROC curves for gastric-type IPMN vs. other neoplasms in U-index of *MUC1*. B: ROC curves for intestinal-type IPMN vs. other neoplasms in U-index of *MUC2*. C: ROC curves for gastric-type IPMN vs. intestinal-type IPMN in U-index of *MUC4*.
(EPS)

Table S1 **Summary of Synthetic oligonucleotides used in the study.**
(XLSX)

Table S2 **Summary of median, 95% confidence interval and P value of KS test in MSE analysis.**
(XLSX)

Table S3 **Summary of sensitivity and specificity in quadratic discrimination analysis and canonical discriminant analysis.**
(XLSX)
